# Damage to the eye and optic nerve in seriously traumatized patients with concomitant head injury: analysis of 84,627 cases from the TraumaRegister DGU® between 2002 and 2015

**DOI:** 10.1186/s13049-020-0712-5

**Published:** 2020-03-02

**Authors:** Torge Huckhagel, Jan Regelsberger, Manfred Westphal, Jakob Nüchtern, Rolf Lefering

**Affiliations:** 1 Department of Neurosurgery, University Medical Center Hamburg-Eppendorf, Hamburg, Germany; 2 Department of Trauma, Hand and Reconstructive Surgery, University Medical Center Hamburg-Eppendorf, Hamburg, Germany; 3 Institute for Research in Operative Medicine (IFOM), University of Witten/Herdecke, Cologne, Germany

**Keywords:** Craniocerebral trauma, Epidemiology, Eye injuries, Optic nerve injuries

## Abstract

**Background:**

*To determine the prevalence and characteristics of prechiasmatic visual system injuries (VSI) among seriously injured patients with concomitant head trauma in Europe by means of a multinational trauma registry.*

**Methods:**

*The TraumaRegister DGU® was searched for patients suffering from serious trauma with a Maximum Abbreviated Injury Scale (AIS) ≥ 3 between 2002 and 2015 in Europe. After excluding cases without significant head injury defined by an AIS ≥ 2, groups were built regarding the existence of a concomitant damage to the prechiasmatic optic system comprising globe and optic nerve. Group comparisons were performed with respect to demographic, etiological, clinical and outcome characteristics.*

**Results:**

*2.2% (1901/84,627) of seriously injured patients with concomitant head trauma presented with additional VSI. These subjects tended to be younger (mean age 44.7* versus *50.9 years) and were more likely of male gender (74.8%* versus *70.0%) compared to their counterparts without VSI. The most frequent trauma etiologies were car accidents in VSI patients (28.5%) and falls in the control group (43.2%). VSI cases were prone to additional soft tissue trauma of the head, skull and orbit fractures as well as pneumocephalus. Primary treatment duration was significantly longer in the VSI cohort (mean 23.3* versus *20.5 days) along with higher treatment costs and a larger proportion of patients with moderate or severe impairment at hospital discharge despite there being a similar average injury severity at admission in both groups.*

**Conclusions:**

*A substantial proportion of patients with head injury suffers from additional VSI. The correlation between VSI and prolonged hospitalization, increased direct treatment expenditures, and having a higher probability of posttraumatic impairment demonstrates the substantial socioeconomic relevance of these types of injuries.*

## Background

Eye injuries are a significant global health problem with a worldwide annual incidence of about 55 million people impaired during their daily activities and 1.6 million people with posttraumatic binocular blindness [1]. The frequency of ocular trauma depends on numerous factors including geographical, socioeconomic and also seasonal circumstances [2–5]. In Germany, annual incidence rates of 302 and 30.5 per 100,000 inhabitants have been determined for mild and severe head trauma previously [6, 7], but there is a general lack of information on the frequency of patients suffering from head and face trauma with additional injury to the prechiasmatic visual system (VSI) which comprises of the globe and the optic nerve. To the best of our knowledge, this is the first-time report aiming to determine the prevalence and characteristics of VSI among seriously injured patients with concomitant moderate to severe head trauma in the Central European setting by means of a large multinational trauma registry.

## Methods

**Fig. 1:**
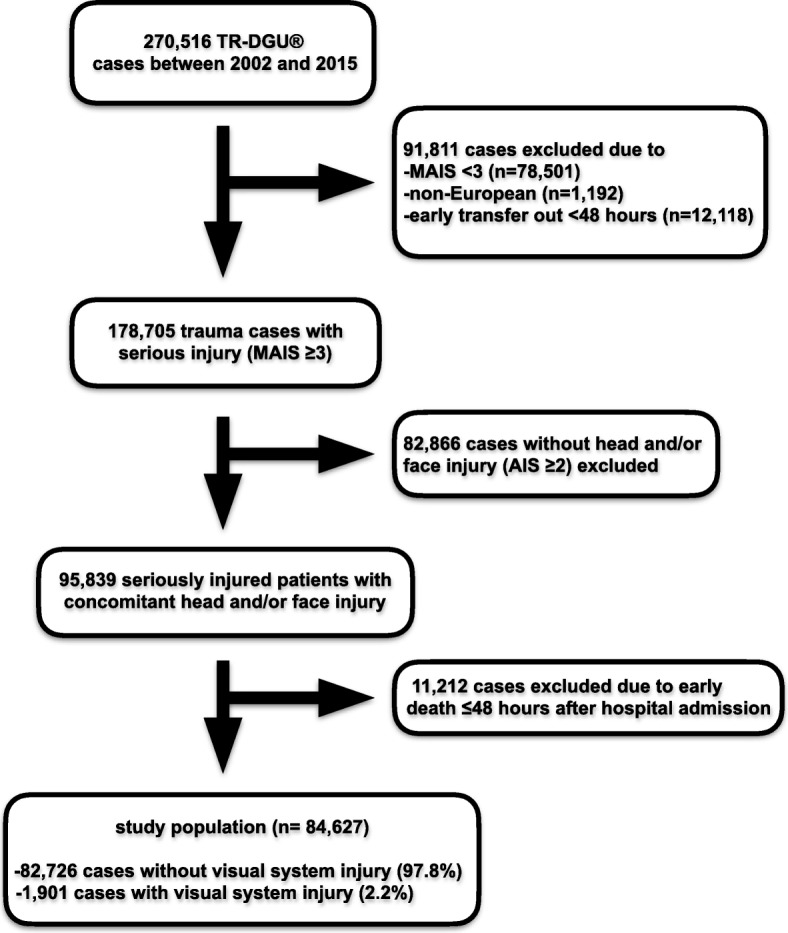
flowchart_study population Description of study inclusion and exclusion criteria. (M)AIS = (Maximum) Abbreviated Injury Scale. TR-DGU = TraumaRegister DGU® of the German Trauma Society.

All data analyzed in this survey were derived from the TraumaRegister DGU® (TR-DGU) upon receipt of an authorization by the institutional review board in 2018. Data presentation follows the established guidelines for reporting observational studies outlined in the strengthening the reporting of observational studies in epidemiology (STROBE) statement [8]. Moreover, the study fully adheres to the publication guidelines of the TR-DGU and is registered as TR-DGU project 2018–014. The TR-DGU of the German Trauma Society (Deutsche Gesellschaft für Unfallchirurgie, DGU) was founded in 1993. The aim of this multicenter database is a pseudonymized and standardized documentation of severely injured patients. The TR-DGU collects data prospectively in four consecutive time phases from the site of the accident until discharge from hospital: A) Pre-hospital phase, B) Emergency room and initial surgery, C) Intensive care unit and D) Discharge. The documentation includes detailed information on demographics, injury pattern, comorbidities, pre- and in-hospital management, course on intensive care unit, relevant laboratory findings including data on transfusion and outcome of each individual. Their inclusion criteria are admission to hospital via emergency room with subsequent intensive care unit (ICU) care or reaching the hospital alive, but with death occurring before ICU admission. The infrastructure for documentation, data management, and data analysis is provided by AUC - Academy for Trauma Surgery (AUC - Akademie der Unfallchirurgie GmbH), a company affiliated to the German Trauma Society. The scientific leadership is provided by the Committee on Emergency Medicine, Intensive Care and Trauma Management (Sektion NIS) of the German Trauma Society. Participating hospitals submit their data, which is pseudonymized, into a central database via a web-based application. Overall completeness of data collection has proven to be high, as laid down in the regularly upcoming reports of the registry [9]. Scientific data analysis is approved through a peer review procedure laid down in the TR-DGU publication guideline. The participating hospitals are primarily located in Germany (90%), but a growing number of hospitals in other countries are contributing data as well. Currently, approximately 35,000 cases from almost 700 hospitals are entered into the database per year. A comprehensive list of all contributing institutions is available at the TR-DGU website (www.traumaregister-dgu.de). Participation in TR-DGU is voluntary. For hospitals associated with TraumaNetzwerk DGU®, however, the entry of at least a basic data set is obligatory for reasons of quality assurance. After identification of a total of 270,516 TR-DGU cases between 2002 and 2015, all patients were assessed according to our study protocol presented in detail in Fig. 1 (flowchart _study population). Of note, all included patients received serious injuries defined by a maximum Abbreviated Injury Scale (AIS) score ≥ 3 and also a collateral head or face trauma with an AIS score ≥ 2. The AIS forms a foundation for several trauma scores including the Injury Severity Score (ISS), which was first published in 1971 and underwent its last update in 2015 (www.aaam.org; accessed 24 April, 2019) [10–12]. Non-European cases were excluded according to our aims to determine VSI epidemiology in the European setting. Patients who died or were transferred to another institution within 48 h after hospital admission were also excluded. This was done for two reasons: 1) Detection of VSI in individuals who died after a short time may be difficult and 2) Patients relocated at an early stage of treatment could otherwise be counted twice erroneously. The resulting study population (*n* = 84,627) was subsequently separated into two groups depending on the existence of an additional VSI. VSI comprise injuries to the globe and/or optic nerve and are represented by the following AIS 2005 codes currently utilized by the TR-DGU: 240499.1, 241,006.2, 241,200.2, 240,402.2 and 240,403.3 for eye injuries of different forms as well as 230,202.2, 230,204.2 and 230,205.3 for traumatic optic neuropathy. Detailed information on the aforementioned codes is provided in supplement 1. VSI were detected by a combined approach including neurological assessment in all cases as well as cranial computed tomography in 91.8% of non-VSI and 93.4% of VSI patients. Both groups (VSI and control group) were compared with regard to demographic and etiological characteristics as well as injury patterns, extent of the trauma measured by the ISS, treatment duration, functional outcome in terms of the Glasgow Outcome Scale Score (GOS) at the end of primary hospital treatment, and need for further inpatient care after primary hospital discharge [13]. Moreover, treatment expenditures were calculated for both cohorts using the TR-DGU cost estimator implemented by Lefering et al. [14]. The registry-based data material is outlined in a descriptive mode with primary focus on practical relevance, because the extensive sample size will easily render even clinically negligible differences statistically significant. Frequencies are presented as percentages with their related 95% confidence intervals (CI) and central tendency measures with their associated standard deviations (SD), where appropriate. All statistical calculations were performed using IBM SPSS Statistics (version 24, International Business Machines Corporation, Armonk, NY, USA).

## Results

**Fig. 2:**
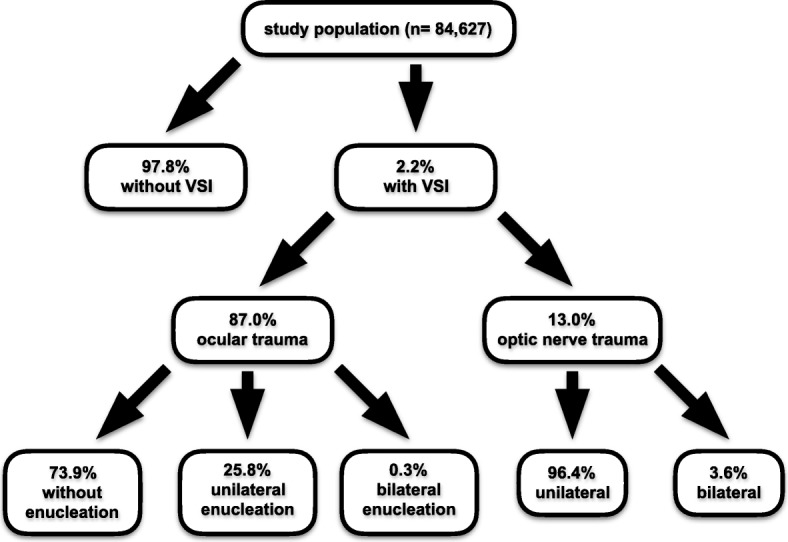
flowchart__distribution of visual system injuries. Composition of the study population with respect to different types of prechiasmatic optic system lesions. VSI = visual system injury

Prevalence of eye and optic nerve injuries in moderate to severe head trauma patients 2.2% (CI 2.1–2.3%) of the total study population (*n* = 84,627) comprised of seriously injured patients with additional moderate to severe head trauma suffered from accompanying VSI. The vast majority of the VSI cases showed ocular damage (87.0%; CI 82.9–91.3%) and only a minority presented with optic nerve trauma (13.0%, CI 11.4–14.7%). One out of four patients with globe injury presented with traumatic loss of an eye and more than 96% of traumatic optic neuropathies were unilateral. The detailed distribution of VSI is delineated in Fig. 2 (flowchart_distribution of visual system injuries).

### Demographic comparison between head trauma patients with and without VSI

VSI patients were more frequently male as compared to the control group (74.8% versus 70.0%). We found that VSI patients were on average 6.2 years younger in comparison to the control group. Table 1 (table_demographic characteristics) provides specific information on demographic characteristics of both cohorts and includes a further differentiation into three distinct age categories.

**Table 1:** Demographic characteristics

	control group	VSI group
**male (%)**	70.0 (CI 69.4–70.6)	74.8 (CI 70.8–79.0)
**mean / median age (years)**	50.9 / 52.0 (SD 22.8)	44.7 / 45.0 (SD 21.4)
**age ≤ 15 years (%)**	4.5 (CI 4.3–4.6)	5.7 (CI 4.7–6.9)
**age 16–59 years (%)**	56.6 (CI 56.1–57.1)	67.3 (CI 63.6–71.1)
**age ≥ 60 years (%)**	38.9 (CI 38.5–39.4)	27.0 (CI 24.7–29.4)

Gender and age distribution of head trauma patients ± concomitant prechiasmatic visual system injury (VSI). *CI* 95% conficence interval, *SD* standard deviation

### Trauma etiology and injury mechanisms

VSI resulted more frequently from car accidents compared to the control group (28.5% versus 18.8%), whereas falls were more commonly seen in the control cohort (43.2% versus 26.6%). Violent attacks including stabbings and shootings were rarely encountered in both groups, but generally affected VSI patients more frequently. Penetrating trauma mechanisms accounted for a slightly larger proportion of VSI compared to head injuries without involvement of the prechiasmatic optic system. Table 2 (table_etiology and mechanism of trauma) reveals in-depth data on trauma etiology and injury mechanisms for both groups.

**Table 2:** Etiology and mechanism of trauma

	control group	VSI group
**traffic accident_car (%)**	18.8 (CI 18.5–19.1)	28.5 (CI 26.2–31.1)
**traffic accident_motorbike (%)**	9.1 (CI 8.9–9.3)	9.1 (CI 7.8–10.5)
**traffic accident_bicycle (%)**	11.7 (CI 11.5–12.0)	11.3 (CI 9.8–12.9)
**traffic accident_pedestrian (%)**	7.7 (CI 7.5–7.9)	6.8 (CI 5.6–8.0)
**high fall > 3 m (%)**	15.3 (CI 15.1–15.6)	12.7 (CI 11.2–14.4)
**low fall < 3 m (%)**	27.9 (CI 27.5–28.3)	13.9 (CI 12.3–15.7)
**punch (%)**	3.0 (CI 2.8–3.1)	6.5 (CI 5.4–7.8)
**shot (%)**	0.3 (CI 0.3–0.4)	1.2 (CI 0.7–1.8)
**stab (%)**	0.3 (CI 0.2–0.3)	0.8 (CI 0.4–1.3)
**other reason (%)**	5.9 (CI 5.8–6.1)	9.2 (CI 8.0–10.8)
**blunt trauma (%)**	97.8 (CI 97.1–98.5)	92.9 (CI 88.6–97.4)
**penetrating trauma (%)**	2.2 (CI 2.1–2.3)	7.1 (CI 5.9–8.4)

Comparison between head trauma cases ± additional prechiasmatic visual system injury (VSI) regarding trauma etiology and underlying mechanism. *CI* 95% confidence interval

### Description of accompanying lesions

VSI patients suffered more frequently from associated thoracoabdominal and limb injuries compared to their non-VSI counterparts, whereas similar frequencies could be recorded for concomitant spine and pelvic trauma. When comparing both groups with regard to intracranial lesions, VSI cases showed larger proportions of primary pneumocephalus (4.1% versus 1.1%) and epidural hematoma (12.7% versus 10.2%), but the frequencies of brain contusion, global brain edema, pituitary and brain stem injuries were distributed almost equally across both groups with pituitary and brain stem lesions being quite rare events. More superficially located damage to the head like soft tissue lacerations and skull fractures - especially orbit and Le Fort fractures - occurred much more commonly in VSI patients compared to the control group. Of note, subdural hematoma and intracerebral hemorrhage were more frequently seen in the control group. Intracranial arteries and veins were injured only sporadically in VSI and non-VSI cases. Table 3 (table_concomitant lesions) delineates comprehensive data on accompanying involvement of other body regions as well as damage to different extra- and intracranial structures for both groups.

**Table 3:** Concomitant lesions

	control group	VSI group
**thorax (AIS > 1) (%)**	43.9 (CI 43.4–44.3)	46.8 (CI 43.7–50.0)
**abdomen (AIS > 1) (%)**	11.9 (CI 11.6–12.1)	15.0 (CI 13.3–16.9)
**spine (AIS > 1) (%)**	25.1 (CI 24.7–25.4)	24.1 (CI 21.9–26.5)
**pelvis (AIS > 1) (%)**	13.1 (CI 12.9–13.4)	13.5 (CI 11.9–15.2)
**upper extremity (AIS > 1) (%)**	28.8 (CI 28.5–29.2)	33.9 (CI 31.4–36.7)
**lower extremity (AIS > 1) (%)**	19.7 (CI 19.3–20.0)	23.4 (CI 21.3–25.7)
**pneumocephalus (%)**	1.1 (CI 1.1–1.2)	4.1 (CI 3.2–5.1)
**brain contusion (%)**	25.2 (CI 24.8–25.5)	26.1 (CI 23.9–28.5)
**global brain edema (%)**	8.9 (CI 8.7–9.1)	9.4 (CI 8.0–10.8)
**pituitary (%)**	0.1 (CI 0.1–0.2)	0.3 (CI 0.1–0.6)
**brain stem (%)**	2.1 (CI 2.0–2.2)	2.3 (CI 1.7–3.0)
**soft tissue of the head (%)**	22.7 (CI 22.4–23.0)	34.8 (CI 32.1–37.5)
**skull fractures (%)**	41.5 (CI 41.0–41.9)	49.2 (CI 46.2–52.5)
**orbit fractures (%)**	10.2 (CI 9.9–10.4)	31.6 (CI 29.5–33.7)
**Le Fort fractures (%)**^**a**^	7.0 (CI 6.8–7.2)	22.4 (CI 20.5–24.2)
**epidural hematoma (%)**	10.2 (CI 9.9–10.4)	12.7 (CI 11.1–14.4)
**subdural hematoma (%)**	33.6 (CI 33.2–34.0)	23.7 (CI 21.5–26.0)
**subarachnoid hemorrhage (%)**	27.3 (CI 27.0–27.7)	26.4 (CI 24.1–28.8)
**intracerebral hemorrhage %)**	15.5 (CI 15.3–15.8)	13.4 (CI 11.7–15.1)
**external carotid artery (%)**	0.0 (CI 0.0–0.0)	0.1 (CI 0.0–0.3)
**internal carotid artery (%)**	0.3 (CI 0.3–0.3)	0.7 (CI 0.4–1.2)
**anterior cerebral artery (%)**	0.1 (CI 0.0–0.1)	0.2 (CI 0.0–0.5)
**middle cerebral artery (%)**	0.2 (CI 0.2–0.3)	0.2 (CI 0.1–0.5)
**posterior cerebral artery (%)**	0.1 (CI 0.1–0.1)	0.2 (CI 0.0–0.5)
**vertebral artery (%)**	0.2 (CI 0.2–0.2)	0.4 (CI 0.1–0.8)
**cerebral veins (%)**	0.4 (CI 0.4–0.5)	0.8 (CI 0.5–1.4)

Detailed exposition of associated lesions in head trauma patients ± prechiasmatic visual system injury (VSI). ^a^ Without Le Fort fracture type 1. *CI* 95% confidence interval

### Injury severity and treatment characteristics

The median ISS was the same in both groups (22.0) indicating a comparable average burden of trauma for VSI and non-VSI cases, but the median number of intubation and ICU days as well as total length of hospital stay varied significantly between both cohorts with a general need for longer median total inpatient treatment time in VSI patients (18.0 versus 15.0 days). In congruence with that finding, estimated median primary care treatment costs of VSI cases were considerably higher compared to head trauma patients without globe or optic nerve injury (16,540.50 versus 13,266.00 Euro). Table 4 (table_severity of injury and treatment duration) gives specific information on injury severity measures, primary care treatment periods and calculated direct medical expenses. 23.4% of globe injuries and 20.5% of optic nerve injuries were treated surgically, but detailed description of the utilized ophthalmosurgical and neurosurgical procedures is well beyond the scope of the TR-DGU and therefore not provided.

**Table 4:** Severity of injury and treatment duration

	control group	VSI group
**mean / median ISS**	23.5 / 22.0 (SD 11.0)	25.1 / 22.0 (SD 10.9)
**preclinical GCS ≤ 8 (%)**	31.2 (CI 30.8–31.6)	30.3 (CI 27.7–33.3)
**preclinical shock (RR sys ≤ 90 mmHg) (%)**	10.4 (CI 10.2–10.7)	14.1 (CI 12.2–16.2)
**mean / median intubation time (days)**	5.8 / 1.0 (SD 9.9)	7.0 / 2.0 (SD 11.0)
**mean / median ICU treatment duration (days)**	10.0 / 5.0 (SD 12.4)	11.7 / 6.0 (SD 13.8)
**mean / median hospital stay (days)**	20.5 / 15.0 (SD 19.7)	23.3 / 18.0 (SD 19.9)
**mean / median calculated treatment costs (Euro)**	21,083.1 / 13,266.0 (SD 21,611.2)	24,511.5 / 16,540.5 (SD 23,825.6)

Severity of injury, duration of primary treatment and estimated medical expenses of head trauma cases ± accompanying injury of the prechiasmatic visual system (VSI). *CI* 95% confidence interval, *GCS* Glasgow Coma Scale, *ICU* intensive care unit, *ISS* Injury Severity Score, *SD* standard deviation

### Outcome following primary treatment

The proportion of patients with mild to severe disability (GOS 3–4) was larger in the VSI compared to the control group (45.1% versus 39.3%), whereas an almost equal percentage of cases with good recovery (GOS 5) could be detected in both cohorts. VSI patients did not differ from non-VSI cases with respect to the need for further inpatient care (e.g. rehabilitation facility) following discharge from the hospital they were admitted to for primary treatment. Table 5 (table_outcome) displays detailed information on GOS scores and hospital discharge status for both groups.

**Table 5:** Outcome

	control group	VSI group
**GOS_1**^**a**^**_death > 48 h (%)**	10.1 (CI 9.9–10.4)	4.7 (CI 3.8–5.9)
**GOS_2_persistent vegetative state (%)**	3.3 (CI 3.1–3.4)	3.0 (CI 2.3–3.9)
**GOS_3_severe disability (%)**	13.1 (CI 12.8–13.3)	16.1 (CI 14.3–18.1)
**GOS_4_moderate disability (%)**	26.2 (CI 25.8–26.5)	29.0 (CI 26.7–31.6)
**GOS_5_good recovery (%)**	47.4 (CI 46.9–47.8)	47.1 (CI 43.9–50.3)
**discharge_home (%)**	42.2 (CI 41.8–42.6)	43.8 (CI 40.8–46.9)
**discharge_rehabilitation (%)**	30.8 (CI 30.4–31.2)	32.9 (CI 30.4–35.5)
**discharge_hospital (%)**	13.7 (CI 13.4–13.9)	15.2 (CI 13.5–17.1)
**discharge_death or other (%)**	13.3 (CI 13.1–13.6)	8.1 (CI 6.9–9.5)

Description of functional outcome for head trauma patients ± prechiasmatic visual system injury (VSI) by means of Glasgow Outcome Scale Score (GOS) and need for further medical attention following primary care discharge. ^a^ Individuals dying later than 48 h following hospital admission. Patients perishing within 48 h after admission were excluded from the analysis according to the study exclusion criteria

## Discussion

We primarily aimed to determine the prevalence and characteristics of VSI in seriously injured patients with concomitant moderate to severe head trauma in Central Europe. According to the TR-DGU data presented in this study, 2.2% of seriously traumatized patients with significant head injury suffer from concomitant VSI with damage to the eye being much more frequent than optic nerve trauma. Recent data published by Maegele et al. reveal an annual incidence of 10.1 moderate to severe head trauma cases per 100,000 inhabitants of Germany, which would result in an estimated incidence of about 0.2 VSI per 100,000 people [15]. The incidence of ocular trauma is known to be dependent on various factors like age, gender as well as socioeconomic and geographical background with a markedly higher risk of eye injuries in developing countries compared to modern industrial societies [3, 16]. Legislative measures, education programs, and the utilization of protection tools have been able to reduce ophthalmic injury rates in children [17–19]. Our finding of globe injury being more frequent than traumatic optic neuropathy is in line with previously published population-based studies [20, 21]. Generally speaking, VSI patients tended to be younger and were more frequently male compared to those without eye or optic nerve damage. Previous studies from other world regions have revealed comparable results with respect to age susceptibility and gender distribution for pediatric and adult eye trauma [5, 22–24]. The vulnerability of adult males with regard to ophthalmic lesions may be related to the higher percentage of male employees in the industrial and agriculture sectors, which are known to be particularly prone to ocular injuries [25]. Trauma etiology was markedly different between patients with versus patients without accompanying VSI. In general, VSI resulted more commonly from car collisions and non-VSI trauma arose more frequently from falls. Injuries due to violent attacks were exceptionally rare, but were more frequently seen in the VSI cohort. The literature reveals heterogeneous information on eye trauma causes. Past surveys reported on a majority of occupation-related eye injuries [21, 23], whereas Qi et al. identified firecrackers and traffic accidents as primary causes of ocular trauma leading to hospitalization in a large Chinese cohort study comprising 5799 patients [26]. Falls were determined to be the leading cause of corneal and scleral ruptures in geriatric patients corresponding to another study from Hong Kong [27]. The discrepancies concerning our results may be explained by the present TR-DGU inclusion criterion of hospital admission via emergency room with subsequent ICU care which deviates from the typical patient population at tertiary eye referral centers without concomitant life-threatening injuries. Our registry-based data reveal more frequent soft tissue lesions and skull fractures including orbit and Le Fort fractures in VSI cases compared to head trauma patients without prechiasmatic visual impairment. A correlation between ocular injuries and orbit fractures has been detected by several authors previously, especially in the case of posterior and/or lateral extension of the fracture [28, 29]. Of note, final visual prognosis is reported to be worse in severe eye trauma with associated orbit fracture [30]. Moreover, frontal bone fractures are known to be closely related to posttraumatic pneumocephalus, which was also seen more commonly in VSI patients in our study [31]. Despite similar global burden of injuries in both cohorts, VSI exhibited longer average ICU and total hospital stay as well as higher primary care treatment expenses. The median treatment duration of 18 days in VSI patients is slightly longer than the average hospitalization time of pediatric eye trauma patients reported by Karim-Zade et al., which might be explained by the associated seriousness of the injuries in the TR-DGU derived cases [32]. More than one out of five VSI patients underwent surgery targeting the globe or optic nerve damage, but detailed information on specific procedures is unfortunately not obtainable from the registry database. There is a growing body of evidence concerning the benefits of early surgical interventions such as globe repositioning and repair, removal of intraocular foreign bodies as well as supporting intravitreal antibiotics and preventive vitrectomy following open globe trauma [3, 33–38]. Patients with VSI showed increased disability rates at hospital discharge compared to those without visual dysfunction. Taking into account the young average age of the VSI patients and the great importance of good vision for most professional activities, one can easily imagine the profound socioeconomic significance of these lesions beyond individual misfortune. On a global scale, Negrel et al. reported on approximately 1.6 million people with injury-related blindness and an additional 2.3 million cases of trauma-related bilateral low vision [1]. One limitation of this study is the lack of information on the type of ocular injury. The TR-DGU is a registry aiming to standardize documentation of severely injured patients and is not explicitly dedicated to eye injuries. Therefore, well-established prognostic scores and specific terminology like the Ocular Trauma Score and the Birmingham Eye Trauma Terminology are not obtainable from the underlying database [39, 40]. Moreover, TR-DGU data collection terminates at hospital discharge, which impedes presentation of long-term functional outcome. All admitted patients are initially examined by specialized trauma surgeons and not primarily by ophthalmologists, which could potentially lead to systematic underreporting of minor ocular injuries. Furthermore, the proportion of patients undergoing dedicated contrast-enhanced vascular imaging is not reported, which may constitute a source of error regarding the detection of accompanying cerebrovascular injuries. Additionally, patients directly transferred to specialized neurosurgical and/or ophthalmological care without trauma team activation upon admission do not meet the inclusion criteria of the registry and therefore constitute a case selection bias. In the early phase of data collection a potential bias resulting from a disproportionate high rate of contributing maximum care hospitals cannot be excluded, but one should keep in mind that patients with head injuries tend to be more likely referred to large trauma centers with neurosurgical service. The possible bias is therefore limited by the inclusion criteria. The major strengths of this survey include its multinational origin, having a large number (more than 84.6 thousand) of head trauma cases, and having a comprehensive evaluation of basic demographics, trauma etiology, associated injuries, treatment course and costs as well as neurologic outcome with respect to present or absent additional VSI. There is a high data representativity and generalizability for Germany due to a nationwide coverage of contributing hospitals of different levels of care. Completeness and quality of data have been shown to meet high standards, as laid down in the official annual reports of the registry [9]. In addition, more than 90% of individuals of both groups underwent emergency cranial computed tomography, which is the preferred imaging tool for the detection of bony skull and facial lesions as well as acute hemorrhages. The complementary approach of clinical and radiological examination provides a substantial increase in the detection rate of damages to the prechiasmatic visual system.

## Conclusion

A substantial number of seriously injured patients with head trauma suffers from additional VSI. Through the provided data concerning characteristic trauma mechanisms and injury patterns, clinicians may be able to better identify and focus on patients at higher risk for ocular and optic nerve lesions, which may eventually improve visual outcomes in these cases. The correlation between VSI and higher probability of posttraumatic functional impairment demonstrates the urgent need for more effective preventive measures as well as improvement of the current therapeutic methods used to treat these injuries in order to reduce their detrimental impact at both the individual and also societal level.

## Supplementary information

**Additional file 1.**

## Data Availability

All epidemiological data presented in this study were retrieved from the TraumaRegister DGU® of the German Trauma Society. The datasets are available from the registry on reasonable request.

## Abbreviations

AIS: Abbreviated Injury Scale
CI: 95% confidence interval
GCS: Glasgow Coma Scale
GOS: Glasgow Outcome Scale
ICU: Intensive care unit
ISS: Injury Severity Score
SD: Standard deviation
TR-DGU: TraumaRegister DGU® of the German Trauma Society
VSI: Prechiasmatic visual system injury comprising globe and optic nerve
