# Supplementary material for: Damage to the eye and optic nerve in seriously traumatized patients with concomitant head injury: analysis of 84,627 cases from the TraumaRegister DGU® between 2002 and 2015
Source: Scand J Trauma Resusc Emerg Med. 2020 Mar 2;28:15. doi: 10.1186/s13049-020-0712-5 (PMC7052966; doi:10.1186/s13049-020-0712-5)
Supplement: Supplementary file 1 — Additional file 1. [file 13049_2020_712_MOESM1_ESM.pdf]

Description of Abbreviated Injury Scale (AIS) 2005 codes regarding globe and optic nerve trauma according to the coding principles implemented by the TraumaRegister DGU® of the German Trauma Society.

240499.1 – Eye injury (not further specified)

241006.2 – Retinal detachment (not further specified)

241200.2 – Sclera laceration / rupture

240402.2 – Eye avulsion / enucleation

240403.3 – Eye avulsion / enucleation (bilateral)

230202.2 – Optic nerve contusion

230204.2 – Optic nerve laceration

230205.3 – Optic nerve laceration (bilateral)
